# Influence of *PICALM* and *CLU* risk variants on beta EEG activity in Alzheimer’s disease patients

**DOI:** 10.1038/s41598-021-99589-y

**Published:** 2021-10-14

**Authors:** Aarón Maturana-Candelas, Carlos Gómez, Jesús Poza, Víctor Rodríguez-González, Vìctor Gutiérrez-de Pablo, Alexandra M. Lopes, Nadia Pinto, Roberto Hornero

**Affiliations:** 1 Biomedical Engineering Group, E.T.S.I. de Telecomunicación, Universidad de Valladolid, 47011 Valladolid, Spain; 2 Centro de Investigación Biomédica en Red en Bioingeniería, Biomateriales y Nanomedicina, (CIBER-BBN), Madrid, Spain; 3 Instituto de Investigación en Matemáticas (IMUVA), Universidad de Valladolid, 47011 Valladolid, Spain; 4 Instituto de Patologia e Imunologia Molecular da Universidade do Porto (IPATIMUP), Porto, Portugal; 5 Instituto de Investigaçāo e Inovaçāo em Saúde (I3S), Universidade do Porto, Porto, Portugal; 6 Centro de Matemática da Universidade do Porto (CMUP), Porto, Portugal

**Keywords:** Genetics, Neuroscience, Neurology, Risk factors, Mathematics and computing

## Abstract

*PICALM* and *CLU* genes have been linked to alterations in brain biochemical processes that may have an impact on Alzheimer’s disease (AD) development and neurophysiological dynamics. The aim of this study is to analyze the relationship between the electroencephalographic (EEG) activity and the *PICALM* and *CLU* alleles described as conferring risk or protective effects on AD patients and healthy controls. For this purpose, EEG activity was acquired from: 18 AD patients and 12 controls carrying risk alleles of both *PICALM* and *CLU* genes, and 35 AD patients and 12 controls carrying both protective alleles. Relative power (RP) in the conventional EEG frequency bands (delta, theta, alpha, beta, and gamma) was computed to quantify the brain activity at source level. In addition, spatial entropy (SE) was calculated in each band to characterize the regional distribution of the RP values throughout the brain. Statistically significant differences in global RP and SE at beta band (*p*-values < 0.05, Mann–Whitney *U*-test) were found between genotypes in the AD group. Furthermore, RP showed statistically significant differences in 58 cortical regions out of the 68 analyzed in AD. No statistically significant differences were found in the control group at any frequency band. Our results suggest that *PICALM* and *CLU* AD-inducing genotypes are involved in physiological processes related to disruption in beta power, which may be associated with physiological disturbances such as alterations in beta-amyloid and neurotransmitter metabolism.

## Introduction

Alzheimer’s disease (AD) is the most prevalent cause of dementia worldwide, with an estimate of over 50 million affected people in 2019^1^. This number is expected to nearly double every 20 years, leading to 152 million by 2050^1^. AD symptomatology is defined by memory loss and cognitive and behavioral dysfunctions. Age and genetics are the most prominent risk factors for developing AD^2^. Among the genetic factors attributed to the disease, amyloid precursor protein (*APP*), presenilin-1 (*PSEN1*), and presenilin-2 (*PSEN2*) mutations have been found to be correlated with early-onset AD^3–5^. On the other hand, late-onset AD is known for being strongly associated with the presence of apolipoprotein E (*ApoE*) $\epsilon$4 allele^2^. Previous research has reported other genetic aspects associated with diverse physiological mechanisms that could contribute to the development of the disease. For instance, alterations in genes regulating autophagy^6^, cell adhesion^7^, endocytosis^8^, or apoptosis^9^ also have an impact on AD progression. One of the most insidious causes of neural disruption is chronic inflammation due to extracellular accumulation of insoluble beta-amyloid peptide (A$\beta$)^10^. Aggregates of A$\beta$ come in the form of neuritic plaques, which have been considered the neuropathological hallmark of AD^2,11^. In fact, confirmation of histologic presence of A$\beta$ is established as one of the main AD biomarkers according to the Aging and Alzheimer’s Association (NIA-AA)^12^. Although the role of A$\beta$ as the origin of the inflammation remains unclear, its relationship with localized neurophysiological disturbances in brain tissue implies to be one of the key aspects in the AD pathogenesis^13^. In this way, evidence of genes participating in A$\beta$-related pathways has been reported. Among them, phosphatidylinositol binding clathrin assembly protein (*PICALM*) and clusterin (*CLU*) genes have been found to be involved in A$\beta$ deposition and clearance^14–17^.

Other widely studied phenomena associated with neurodegeneration are changes in the behavior of neurotransmitter neuronal populations. Particularly, AD is characterized by significant alterations in neurotransmitter balance. Acetylcholine and glutamate deficits have been previously associated to AD progression, since they may play key roles in cognitive impairment^18^. Acetylcholine and glutamate neurotransmitters are intimately involved in memory and learning processes, in which they exhibit functional synergy in neuronal firing of pyramidal neurons^18^. However, many other neurotransmitter systems, such as GABAergic, serotonergic, and possibly dopaminergic neurons, have been associated with AD abnormalities^19^. The importance of these aspects in the understanding of dementia are apparent, as new methods of AD diagnosis have been developed measuring these biochemical alterations^20^, along with therapy strategies targeting these deficits modulating neurotransmitter expression via drug administration^21^. Neurotransmission functionality has also been related to genetic features. For instance, *PICALM* and *CLU* have been suggested to be implicated in several neurotransmission mechanisms^22–24^. These relationships imply that alterations in the genetic expression could manifest in synaptic disturbances. In addition to the evidence linking *PICALM* and *CLU* to neurochemical processes, these genes have also been associated with AD through genome-wide association studies^25,26^. Single nucleotide polymorphisms (SNP) rs3851179 and rs11136000 showed correlation with reduced risk of late-onset AD^25,26^. Afterwards, the association between the aforementioned SNPs and the risk of late-onset AD was confirmed^27^.

Another interesting approach to investigate the neurodegeneration associated to AD is the examination of alterations in functional brain activity. For this purpose, the analysis of electroencephalographic (EEG) activity has been extensively used. EEG can keep track of the rapid and transient nature of neuroelectric dynamics due to its high temporal resolution. This technique comes with advantages like low cost, portability, and non-invasiveness. The usefulness of EEG signal analyses has been demonstrated in the study of brain activity disruptions caused by AD^28,29^. However, EEG measurements are acquired on the scalp surface, being unable to illustrate the activity and location of individual neuronal oscillators and thus identify aberrant neural behavior in specific brain areas. To overcome this limitation, source localization procedures have been developed. Standardized low resolution electromagnetic tomography (sLORETA) is based on the assumption that neighboring neurons are strongly correlated, yielding zero localization error in the simulations^30^. Brain activity alterations in AD have been broadly studied analyzing source-level signals acquired by sLORETA. For instance, cortical sources of resting-state EEG were assessed in mild AD patients, obtaining a power increase of widespread delta sources and a power decrease of posterior alpha sources^31^. Furthermore, functional connectivity was investigated including deeper regions of the brain; the results revealed that AD networks were associated with lower global information processing and higher local information processing^32^. Previous research indicated a correlation between source-level EEG alterations and presence of *PICALM* and *CLU* risk alleles. Ponomareva *et al*.^33^ investigated the EEG from healthy subjects and obtained higher power in alpha-3 band (10.9–12.9 Hz) in the *CLU* CC allele group, in addition to higher beta power and lower alpha inter-hemispheric connectivity in the *PICALM* GG allele group^34,35^. These insights reveal an association between abnormal power fluctuations and certain SNPs in *PICALM* and *CLU*, which could be linked to dysfunctions caused by higher A$\beta$ accumulations or disrupted neurotransmission mechanisms, even in pre-clinical stages of the disease^33–35^. However, to the best of our knowledge, no assessment of multiple EEG sources under the effects of both *PICALM* and *CLU* risk alleles in AD patients has been conducted before. Thus, the novelty of this study is the investigation of the relationship between source-level EEG and AD-inducing genotypes associated with shared aspects of neurochemical metabolism.

As suggested by the aforementioned evidence, the relationship between AD-inducing variants of *PICALM* and *CLU* and neuronal dysfunctions are mainly twofold. First, the association with impaired performance of A$\beta$ clearance, and thus to greater inflammatory responses that could affect neurodegeneration. Second, the implications in primary neurotransmitter balance, which could lead to abnormal synaptic behavior. Therefore, we hypothesize that the presence of these alleles (rs3851179 and rs11136000 SNPs) may have an impact on brain activity. Consequently, this study is aimed at analyzing source-level EEG in AD patients and healthy controls with risk and protective alleles of *PICALM* and *CLU* in order to detect which alterations of brain electrical dynamics could be potentially connected to localized A$\beta$ presence and neurotransmission deficits.

## Methods

### Subjects

Fifty-three AD patients (16 males and 37 females) and 24 healthy controls (13 males and 11 females) were enrolled in this study. AD patients met the diagnosis criteria according to the NIA-AA^36^. Additional inclusion and exclusion conditions were applied to participate in the study. Inclusion requisites were ages older than 65 and presence of risk or protector alleles for both rs3851179 and rs11136000 simultaneously. Exclusion conditions included clinical history of neoplasia (active or under treatment), recent surgery, hypercatabolic states, or vascular pathology. Also, cases with atypical signs in the evolution of the disease in AD patients or chronic alcoholism were disregarded. *PICALM* risk and protective genotypes were established as the presence of GG and AG+AA genotypes, respectively. In the case of *CLU*, risk and protective alleles were CC and CT+TT. These decisions were made consistently with previous research assessing the impact of *PICALM* and *CLU* alleles that confer risk or protection against AD^33,34^. Evaluation of cognitive state was performed for each subject using Mini-Mental State Examination (MMSE)^37^. Informed consent was acquired from each subject, relative or legal representative of each subject, in line with the recommendations of the Code of Ethics of the World Medical Association. The study was in compliance with the Declaration of Helsinki. The study protocol was approved by The Ethics Committee at the Porto University (Porto, Portugal. Report n$^{\circ }$ 38/CEUP/2018). Demographic data is presented in Table 1. Statistical analyses were performed to identify potential confounding factors using $\chi ^{2}$ test ($\alpha$ = 0.05). Subjects were matched by sex ($\chi ^{2}_{CON}$(1, *N* = 24) = 1.51, *p*-value = 0.219; $\chi ^{2}_{AD}$(1, *N* = 53) = 1.23, *p*-value = 0.266), and *ApoE* $\epsilon$4 presence ($\chi ^{2}_{CON}$(1, *N* = 22) = 0.386, *p*-value = 0.534; $\chi ^{2}_{AD}$(1, *N* = 49) = 1.34, *p*-value = 0.249). Regarding AD subgroups, patients were matched by severity ($\chi ^{2}$(2, *N* = 53) = 2.65, *p*-value = 0.265). Also, no statistically significant differences between genetic groups were found considering the age or MMSE score of the subjects (*p*-values > 0.05, Mann–Whitney *U*-test). AD severity was adjusted to Reisberg scale criteria^38^ according to 3 severity stages: mild AD ($\text {AD}_{\text{MIL}}$), moderate AD ($\text {AD}_{\text{MOD}}$), and severe AD ($\text {AD}_{\text{SEV}}$). All the comparisons showed no evidence of statistical dependency of any factor towards presence of risk or protective alleles.

**Table 1:** Demographic data.

Genotype	Group	N	Age (mean ± SD) (years)	Sex (M:F)	MMSE (mean ± SD)
Risk alleles	Healthy controls	12	80.33 ± 6.64	8:4	29.33 ± 0.65
	$\text {AD}_{\text{MIL}}$ patients	6	80.00 ± 4.38	2:4	21.00 ± 1.26
	$\text {AD}_\text{MOD}$ patients	8	84.25 ± 3.24	2:6	12.50 ± 1.60
	$\text {AD}_{\text{SEV}}$ patients	4	76.50 ± 7.94	0:4	2.25 ± 4.50
Protective alleles	Healthy controls	12	81.41 ± 7.75	5:7	28.59 ± 1.08
	$\text {AD}_{\text{MIL}}$ patients	17	79.29 ± 8.42	9:8	23.06 ± 2.75
	$\text {AD}_{\text{MOD}}$ patients	8	83.13 ± 6.94	0:8	14.13 ± 2.30
	$\text {AD}_{\text{SEV}}$ patients	10	80.70 ± 5.08	3:7	4.40 ± 4.27

SD, standard deviation; M, male; F, female; $\text {AD}_{\text{MIL}}$, mild AD; $\text {AD}_{\text{MOD}}$, moderate AD; $\text {AD}_{\text{SEV}}$, severe AD; MMSE, Mini-Mental State Examination score.

### Genetic analysis

A sample of saliva from each subject was collected by means of a DNA Genotek Oragene DNA kit (OG-500). The biological samples were genotyped using Thermo Fisher Scientific Axiom$^{\text{TM}}$ Spain Biobank Arrays at the Spanish National Center for Genotyping (CeGEN, Santiago de Compostela, Spain), and SNP calling was performed with Affymetrix Power Tools.

Variant calling quality control (QC) was implemented in the genotyping. This protocol was followed by both individual and marker analyses, according to Affymetrix best practices guide. Criteria of variant calling QC invalidates subjects with dish QC or QC call rates below the defined thresholds. Also, subjects with heterozygosity rates greater than the defined acceptance threshold were not considered for further analysis. *PICALM* and *CLU* risk variants (rs3851179 and rs11136000) qualified as valid in the QC analysis.

### EEG recordings and pre-processing

For each subject, five minutes of resting-state EEG data were recorded with a 19-channel Nihon Kohden Neurofax JE-921A EEG System at electrodes F3, F4, F7, F8, Fp1, Fp2, T3, T4, T5, T6, C3, C4, P3, P4, O1, O2, Fz, Cz, and Pz of the international 10–20 system. The sampling frequency was set to 500 Hz. During acquisition, common average reference was applied. Participants were asked to stay relaxed in a noise-free environment along the procedure. The state of vigilance was controlled by the researchers to prevent drowsiness.

EEG data were stored as ASCII files in a personal computer. Signals were preprocessed according to previous studies^39,40^: (i) mean removal; (ii) 50 Hz notch filter; (iii) Hamming-window bandpass filter between 1 and 70 Hz; (iv) independent component analysis (ICA) to remove components associated with other biosignals; (v) segmentation into epochs of 5 s duration; and (vi) visual rejection of epochs containing artifacts. The average number of artifact-free epochs selected per subject was 39.95 ± 12.74 (mean ± SD).

After EEG acquisition, source-level activity was estimated using sLORETA source localization algorithm^30^. sLORETA computes 3D linear solutions for the EEG inverse problem, allowing a noise-normalized estimation of the distribution of electrical activity within a brain model. An identity matrix was used as noise covariance, as no noise recordings were available. A total of 68 brain regions of interest (ROI) were defined according to the Desikan-Killiany atlas, a gyrus-based classification of regions corresponding to their dominant function^41^. Digital pre-processing and analysis of the signals were carried out with MATLAB$^{\circledR }$ (R2018a version, Mathworks, Natick, MA) and Brainstorm toolbox (documented and freely available for download online under the GNU general public license, http://neuroimage.usc.edu/brainstorm^42^).

### EEG processing

Relative power (RP) was calculated for each artifact-free epoch for the 68 ROIs proposed in the Desikan-Killiany atlas^41^. RP is a commonly used metric to estimate the relative contribution of power of each frequency band to the global power spectrum of a signal^43^. RP has relatively low inter-subject variability and provides power estimations that do not depend on the filtering and amplification characteristics of the recording device^43^. RP equation is described below:

1 $$\begin{aligned} RP_{band} = \sum _{f \in f_{p}}PSD_{n}(f), \qquad f_{p} = \{\delta , \theta , \alpha , \beta , \gamma \}, \end{aligned}$$

where *PSD* is the normalized power spectral density, associated with each frequency component *f*. RP was computed in the traditional frequency bands: delta ($\delta$, 1–4 Hz), theta ($\theta$, 4–8 Hz), alpha ($\alpha$, 8–13 Hz), beta ($\beta$, 13–30 Hz), and gamma ($\gamma$, 30–70 Hz). Once RP values were obtained for each artifact-free epoch, they were averaged for each ROI, obtaining an array of 68 values per subject and frequency band.

In order to assess the heterogeneity of the distribution of RP values, spatial entropy (SE) was calculated. This parameter is based on the Shannon definition of entropy. Homogeneous distributions result in low values of SE, and vice versa. SE was calculated from the RP values from the 68 ROIs for each frequency band in each artifact-free epoch. Then, the averaged SE along epochs was calculated. SE is defined as follows:

2 $$\begin{aligned} SE_{band} = \frac{1}{logN}\sum _{n}^{N}PDF_{n}(f_{p})log(PDF_{n}(f_{p})), \qquad f_{p} = \{\delta , \theta , \alpha , \beta , \gamma \}, \end{aligned}$$

where *n* is each bin in the probability density function (*PDF*), and *N* is the total number of bins. The probability density function was estimated by means of the normalized histogram of RP values at each frequency band. The Freedman-Diaconis rule was used to set the number of bins of the histogram^44^.

### Statistical analysis

Initially, we carried out an exploratory analysis to evaluate normality and homoscedasticity of RP distributions at each frequency band. For this purpose, Kolmogorov–Smirnov and Levene tests were conducted. Nor RP neither SE values met parametric assumptions; therefore, statistical differences between risk and protective alleles subgroups were calculated with Mann–Whitney *U*-tests ($\alpha$ = 0.05). In addition, a false discovering rate (FDR) controlling procedure was used to deal with multiple comparisons assessing differences for each ROI^45^.

## Results and discussion

### Potential biochemical implications of *PICALM* and *CLU* risk variants and their relationship with RP alterations

**Figure 1:**
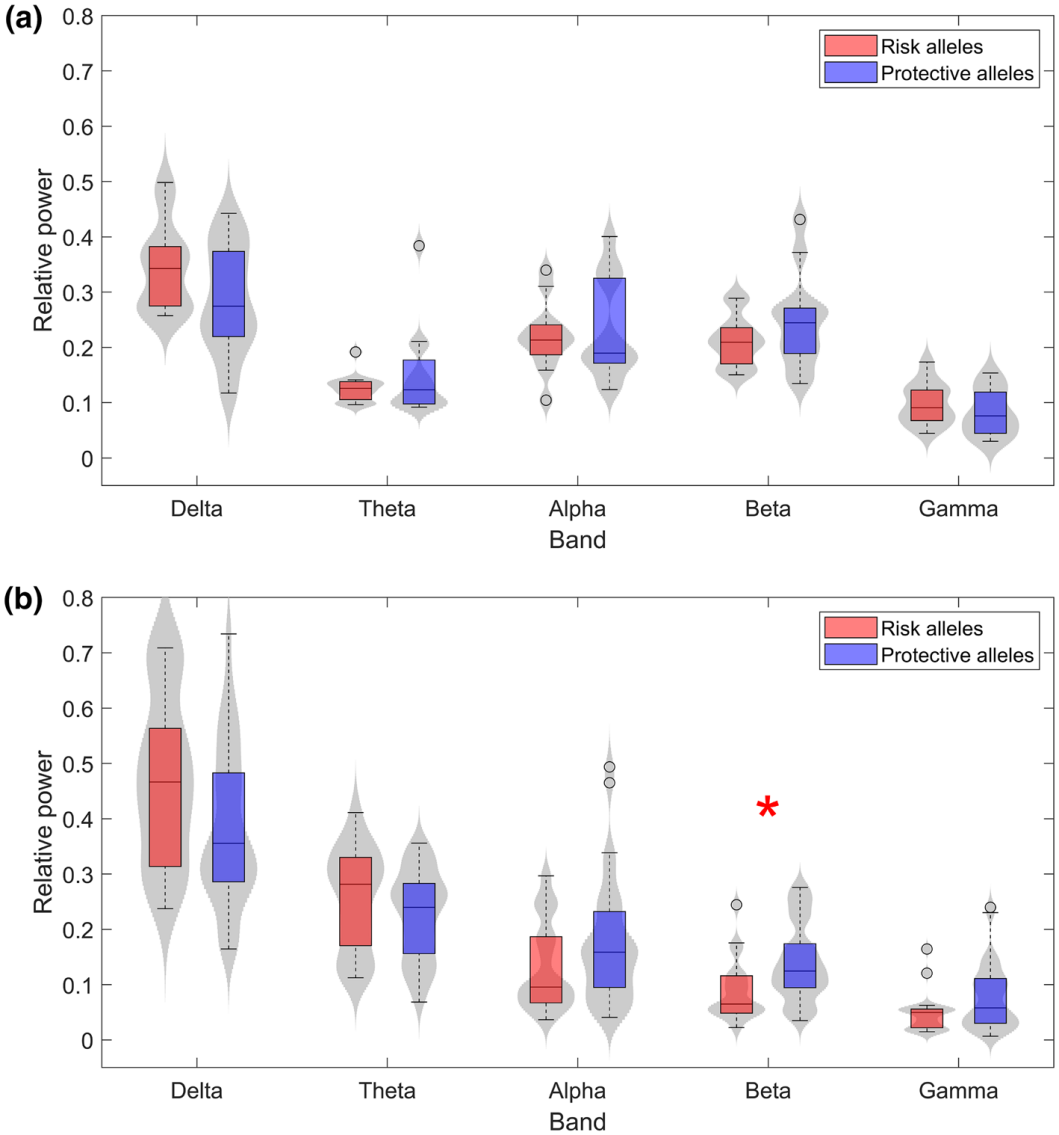
Grand-averaged RP values across ROIs for (**a**) controls and (**b**) AD patients in each frequency band. Subjects with risk alleles for both *PICALM* and *CLU* are represented in red, whereas subjects with both protective alleles are displayed in blue. Statistically significant differences (*p*-value < 0.05, Mann–Whitney *U*-test) are highlighted with a red asterisk.

RP in each ROI was calculated for 53 AD patients (18 with risk alleles of both *PICALM* and *CLU* genes and 35 with the protective variants) and 24 healthy controls (12 with risk alleles and 12 with protective alleles) in the traditional frequency bands. Figure 1 displays the distribution of the grand-average RP values of all ROIs in both control (1.a) and AD patients (1.b). In AD, the risk group showed higher RP values in lower frequency bands (delta and theta) and lower RP values in higher frequency bands (alpha, beta, and gamma). Only significant statistical differentiation between risk and protective genetic variants was found in beta frequency band (*U*-value $=$ 342, *p*-value $=$ 0.007, Mann–Whitney *U*-test). No other band showed statistically significant differences, although delta band was close to the limit of statistical significance (*U*-value $=$ 584, *p*-value = 0.067, Mann–Whitney *U*-test). In general, signs indicating slowing of the EEG have been commonly identified as a quantitative manifestation of neurodegeneration^46^. Interestingly, the observed disturbances in each single frequency band are in line with the typical spectral alterations found in dementia^46^, which suggests that risk genotypes may be associated with worse cognitive status. Given that statistical analyses indicated no evidence of significant confounding factors, alterations of EEG global spectral features are influenced most likely by the genetic AD-inducing effects of the proposed SNPs, and not by an AD severity bias.

On the other hand, the control group exhibited slightly different trends in alpha and gamma. Beta band kept showing higher RP values in the protective genotype, however, significant differences were not obtained. This finding may be due to the opposite influence of *PICALM* alleles in healthy subjects, which has been previously reported^34^. This new insight suggests that the effect of *PICALM* gene could differ depending on the cognitive status of the subject or, at least, on the severity of neurodegeneration. Beta band has been previously associated with not only sensory-motor processing^47^, but also with cognitive processes like spatial and temporal orienting of attention^48^ or working memory^49^. Previous evidence reported that excess of amplitude in beta is associated with stress, anxiety, overthinking, and overstimulation^50^. On the other hand, the hallmark of neurophysiological rhythms alterations in AD is a general slowing of the EEG^46^, in which beta activity is significantly reduced^51,52^. These points altogether lead to consider that certain ranges of EEG beta power could be associated with better cognitive performance. Since *PICALM* GG genotype carriers have been previously suggested to be prone to stress reactions^34^, we could hypothesize that this version of *PICALM*, in general, tends to destabilize neural dynamics under abnormal conditions. In other words, the protective allele of *PICALM* may be associated with more stable ranges of beta activity, closer to healthier brain functioning. This reasoning could also be applied to other frequency bands. For instance, alpha band exhibited a change of tendency in control RP values in comparison with AD. Although no statistically significant differences were observed, this finding may be another case of different effects of the genotype on the EEG dynamics depending on the status of neurodegeneration. A previous study reported an association between *CLU* and RP in upper alpha band for healthy cognitive subjects^33^, which presented higher values for the risk allele. Alpha oscillations reflect neural processing related with essential cognitive functions, such as memory, intelligence, and attention^53,54^. However, excessive values of alpha power have been associated with cholinergic network alterations that could affect synaptic efficacy and reduce cortical connectivity^55^. Thus, the tendency change observed in our results may be due to significant disturbances on physiological mechanisms involving neurotransmitter populations. In this way, *CLU* protective allele could be implicated in cholinergic endeavors closer to healthy conditions in any cognitive status.

**Figure 2:**
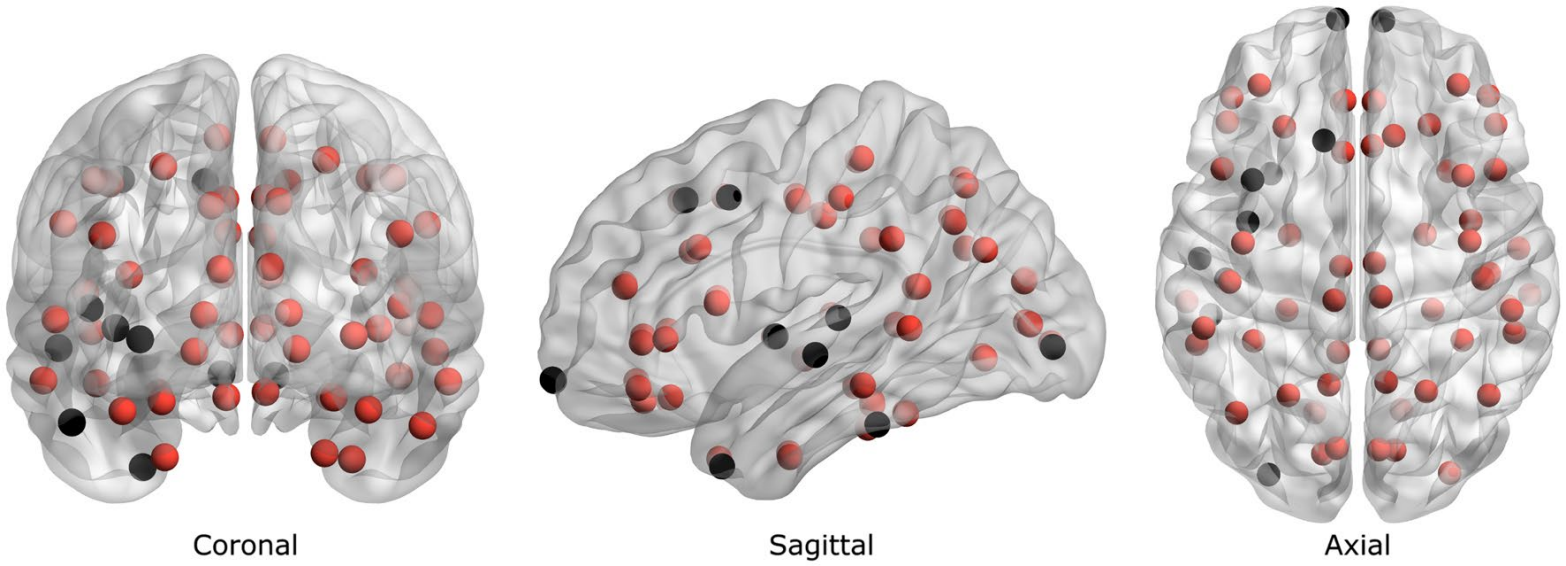
Brain model representing localizations of each ROI according to the Desikan-Killiany atlas. Red balls represent ROIs that showed statistically significant differences in beta RP values between AD patients with risk and protective *PICALM* and *CLU* alleles (FDR-corrected *p*-value < 0.05, Mann–Whitney *U*-test).

Given that *PICALM* and *CLU* genes seem to be related with A$\beta$ clearance processes, we suggest that malfunction of these mechanisms may have some impact in establishing physiological conditions for greater A$\beta$ deposition in the brain, and hence, amplify the detrimental effects of dementia. In this study, statistically significant differences in RP values at beta band were reported in 58 out of the 68 ROIs in AD (FDR-corrected *p*-values < 0.05, Mann–Whitney *U*-test), and no differences in any ROI at all in any frequency band for controls. The obtained differences in AD were represented in a 3D brain model (BrainNet Viewer, v1.63^56^) in Fig. 2, which exhibited beta RP disruptions throughout the brain. According to previous research investigating histological alterations in AD, no spatial preference was observed in the deposition of neuritic plaques in affected brains^57^. In fact, the distribution of neuritic plaques presented large between-subject variation, being opposite to what was found regarding with neurofibrillary tangles^57^. This study assumes that *PICALM* and *CLU* risk variants may stimulate A$\beta$ deposition hindering A$\beta$ clearance mechanisms, since these genes may be involved in these processes^14–17^. Given that A$\beta$ is broadly distributed across the brain in AD, and EEG disturbances in patients with risk alleles of both *PICALM* and *CLU* are also obtained in most ROIs, it can be proposed that the reported EEG abnormalities may be associated with increased accumulation of A$\beta$. Noteworthy, all the regions that did not show evidence of significant differences were found in the left hemisphere, except a single ROI located in the right frontopolar lobe. This observation should be cautiously interpreted, since *p*-values obtained in the spatial analysis were close to the significance threshold ($\alpha$ = 0.05). This finding may not be due to the distribution of A$\beta$, but to the functional asymmetry of the brain. In fact, left hemisphere and beta band activity have been reported to be preferentially engaged in time perception^58–60^, a cognitive process that could be heavily present during resting-state, while the patient is waiting till the end of the EEG acquisition. Slight variations in beta activity caused by functional specialization of the brain may be the reason why some ROIs are not expressed as significantly different by the statistical analyses.

Apart from its inflammatory effect^10^, A$\beta$ may have a significant impact on neurophysiology on additional scales. For instance, the implications of greater presence of A$\beta$ in synaptic functionality have been previously examined. Several studies connecting dopamine metabolism with A$\beta$ deposition were reported, obtaining a decreased release of this neurotransmitter in A$\beta$-infused rat brains^61,62^. Thus, we suggest that *PICALM* and *CLU* risk variants (and hence greater accumulations of A$\beta$ in brain tissue) may be closely related to EEG alterations in beta band caused by dopaminergic deficits. Also, the relation between A$\beta$-mediated inflammation and neurotransmitter disturbances has been assessed, obtaining significant interactions with cholinergic, serotonergic, and dopaminergic neuronal populations^63^. However, confirming direct causality between AD-inducing genotypes and neurotransmission alterations caused by A$\beta$-related pathological conditions, such as chronic inflammation, requires further investigation.

### Alterations on RP distributions and their potential relationship with AD as a disconnection syndrome

**Figure 3:**
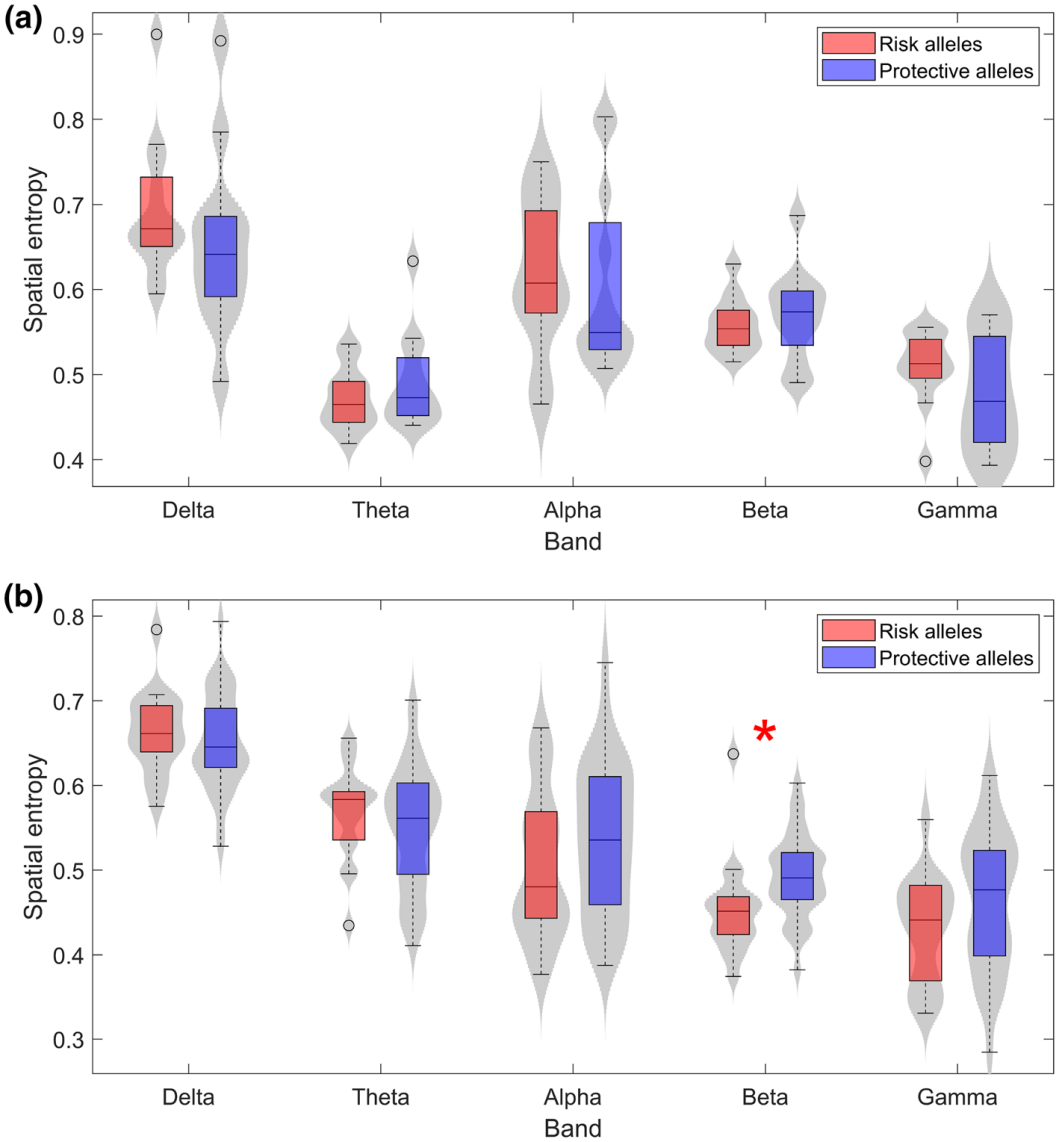
Spatial entropy of RP values for (**a**) controls and (**b**) AD patients in each frequency band. Subjects with risk alleles for both *PICALM* and *CLU* are represented in red, whereas subjects with both protective alleles are displayed in blue. Statistically significant differences (*p*-value < 0.05, Mann–Whitney *U*-test) are highlighted with a red asterisk.

The homogeneity of the RP values throughout the brain was assessed by applying the spatial entropy to RP values. Figure 3 displays the distribution of the SE values in each frequency band in controls (Fig. 3a) and AD patients (Fig. 3b). In AD, the analysis exposed higher values of entropy in subjects with protective alleles in alpha, beta, and gamma bands, and lower values in delta and theta bands. Furthermore, beta band revealed statistically significant differences between subjects with risk and protective alleles in the AD group (*U*-value $=$ 321, *p*-value $=$ 0.002, Mann–Whitney *U*-test). As specialized cortical areas working interdependently were previously identified^64^, entropy alterations could be an effect of impaired communication between different neural populations. The synchronous activation of these neural networks to perform complex processes may result in electrical activity of diverse power and frequencies. Additionally, it has been previously proposed that higher frequencies build local patches of synchrony, while slower rhythms are more present in long-distance interactions^65,66^. Previous research investigated these inter-neural synergies by means of EEG coherence analyses while assessing intelligence performance^67^. In this study^67^, lower coherence results were obtained in higher IQ subjects, which is suggested to be associated with increased neural efficiency and brain complexity. Since coherence is defined by the spectral correlation between two time series or, in other words, how likely a series could be predicted by another one, low coherence values are expected to be related with high SE values and vice versa. These assumptions are consistent with a model that relates lower coherences to increased spatial differentiation and hence, to higher processing efficiency^68,69^. As higher frequencies may be more sensitive to changes in the spatial organization of specialized neural populations, it seems reasonable that RPs in higher frequency bands could be more affected by physiological disturbances that alter connectivity, albeit in a subtle way, since they work locally, influencing specific and smaller neighboring neural populations. On the other hand, Fig. 3a shows the SE values obtained for the control group. The significant differences found in beta for AD patients were not obtained. Furthermore, SE trends in controls changed from AD similarly to what was observed in RP. This finding could indicate that, analogously with RP, SE values in certain intervals may be associated with optimal brain functionality. Also, protective alleles of *PICALM* and *CLU* could participate in maintaining SE in those ranges. To conclude, our SE results in beta band suggest a possible association between AD patients with *PICALM* and *CLU* risk alleles and a worse cognitive functioning.

### Impact of *PICALM* and *CLU* risk alleles on the EEG spectral properties and neurotransmitter metabolism along the AD continuum

**Figure 4:**
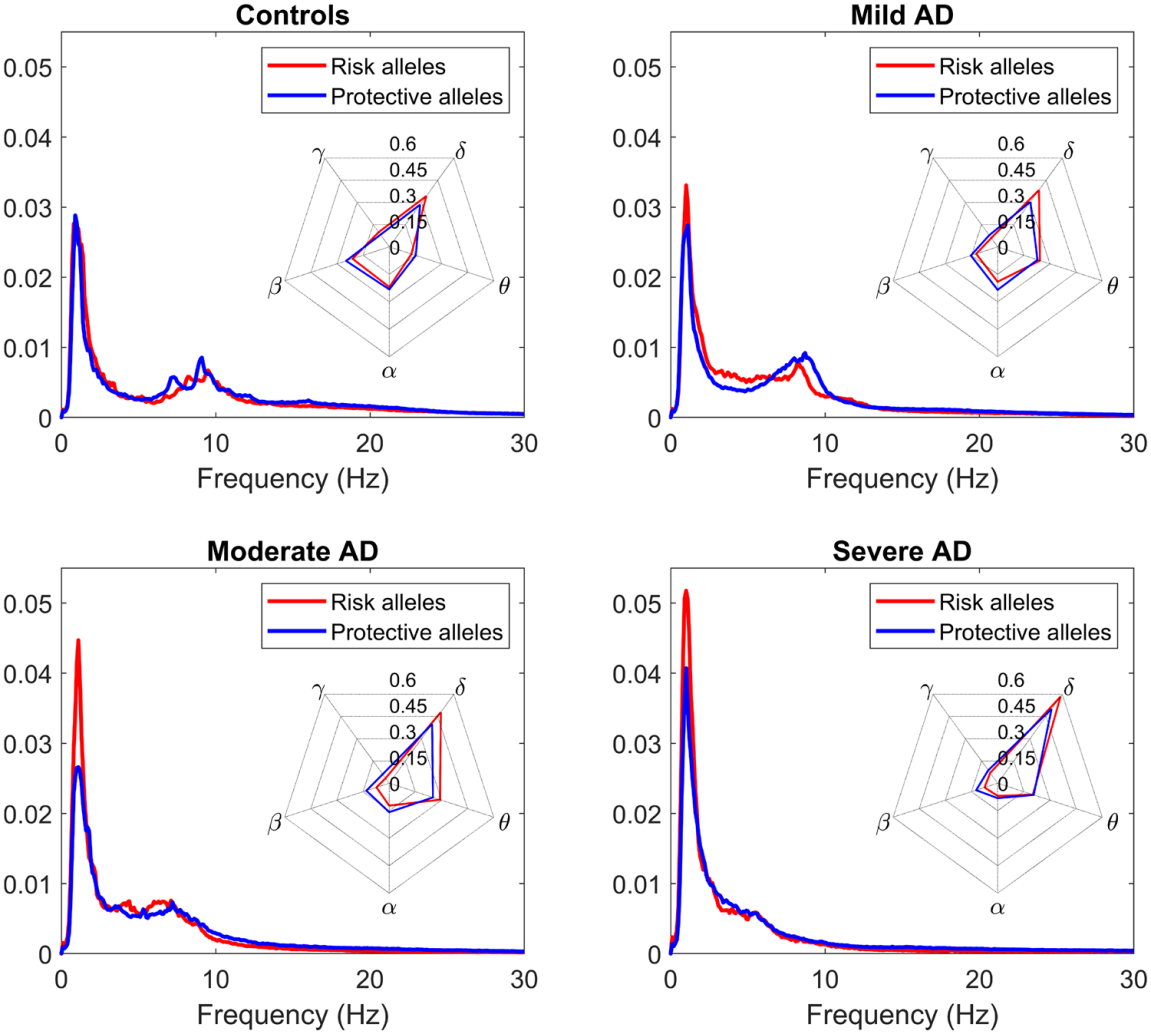
Normalized PSDs for controls and AD patients at different stages of the disease. The grand-averaged spectra are displayed from subjects with risk and protective alleles. Spider plots presenting RP values at each frequency band are shown for the four groups (Controls, Mild AD, Moderate AD, and Severe AD).

Finally, PSDs were inspected along the AD continuum. AD patients were organized corresponding to the stage of the disease: mild, moderate, or severe, according to the Reisberg scale^38^. PSDs were displayed for controls and AD patients at each stage of the disease (Fig. 4), which showed a general slowing of the EEG in the risk group compared to the protective one. Slowdowns in the EEG have been previously associated with disruption of information processing and cognitive dysfunction^46^. Hence, our results suggest that patients with both *PICALM* and *CLU* risk alleles have a deterioration status equivalent to more severe stages of neurodegeneration. Indeed, the normalized PSD from mild AD subjects with the risk alleles is apparently more similar to the PSD from moderate AD subjects with protective alleles than to their protective counterpart. Furthermore, PSDs showed greater differences between genotypes in the mild AD group than in the severe AD group; the EEG slowdown is more evident between $\hbox {AD}_{\text{MIL}}$ patients subgroups (subgroup with risk alleles and subgroup with carriers of protective alleles) than between $\hbox {AD}_{\text{SEV}}$ patients subgroups. This observation suggests that the effects of *PICALM* and *CLU* risk alleles may be more notorious in earlier stages of the disease. In fact, cytopathology in cortical cholinergic pathways has been found to be an early event in the AD continuum^70^. As the genes under study may be involved on neurotransmitter disturbances, the presence of risk alleles could lead to a greater impact in early AD. On the other hand, given the correlation between EEG spectral alterations throughout the brain and A$\beta$ presence, and the common implication of these genes in A$\beta$ modulation^14–17^, we suggest that increased A$\beta$ accumulation may be related to the disruption of spectral dynamics. In addition, the presence of AD-inducing *PICALM* and *CLU* variants seems to have a stronger effect on beta power.

Beta frequency band also showed consistently lower RP values for risk-genotype subjects along the AD continuum. Besides, differences in beta band are preserved as severity increases, while they decrease in theta, alpha, and gamma. This observation suggests the persistent effect that AD-inducing genotypes cause to neural dynamics along the disease progression. This issue may be due to augmented expression of the implicated genes in the last stages of the AD. In this line, previous research observed plasma clusterin levels correlating positively with deterioration status^71,72^. In addition, the brain dopaminergic system was found to be affected by AD, decreasing the ability of dopamine reuptake, and correlating with the severity of the extrapyramidal symptoms^73^. The significant differences observed in beta band may be a manifestation of other neurotransmitter abnormalities. In a previous study, a loss of beta synchronization was found in the early stages of AD development, but not in other frequency bands^74^. Connectivity metrics allow evaluating how local specialized neural networks work together to integrate information. Beta-specialized neuronal populations may become isolated due to alterations in anatomical pathways and, therefore, a decrease in RP at beta band may be a reasonable effect of this aspect. However, if AD is identified as a disconnection syndrome from the perspective of neuronal loss exclusively, alterations in a single frequency band would be difficult to explain^74^. For this reason, other factors that contribute to loss of synchrony, such as cholinergic disturbances, may be relevant. In fact, blockade of the cholinergic system has previously been associated with reduced coherence of the EEG at rest^75^. Given the association of *PICALM* and *CLU* with different neurotransmitter metabolisms^22–24^, carriers of AD-inducing alleles may be expressing these physiological anomalies in the form of RP changes at beta band. To sum up, disruptions in several neurotransmitter systems may play a crucial role in the EEG alterations due to *PICALM* and *CLU* risk genotypes.

## Conclusion

In the present study, an association between AD-inducing *PICALM* and *CLU* alleles and disturbances in the EEG was revealed. Despite our outcomes being relevant, some methodological issues should be taken into account. First, although our results indicate a potential relation between A$\beta$ accumulation or neurotransmitter deficits and alterations in beta EEG activity, these associations are merely speculative. Unfortunately, we have no access to medical imaging data to validate causal relationships between our results and alterations in neurochemical metabolism. For this reason, this study is not so much about ascertaining the associations between alleles and EEG alterations, but rather to exhibit possible physiological causes related to these alleles that may be associated to neurodynamic disturbances. Hence, there is the possibility that other underlying factors may be causing these changes as a hidden side effect of the *PICALM* and *CLU* alleles. In order to clarify the main objective of this article, the associations reported here are a summary of all the potential biochemical causes involving *PICALM* and *CLU* after an extensive investigation of the literature. A main part of the study is the elucidation of how these aspects are potentially related to beta power in brain activity. However, finding causality between the proposed physiological elements and EEG perturbations will be planned in subsequent research projects, in which we intend to have medical imaging available. Obtaining information on the hallmark brain changes of AD will allow verifying the results showed in this study.

In addition, due to the highly restrictive genetic conditions to participate in the study (AD patients and controls with a pair of either risk or protective variants), the number of selected participants became relatively small. In order to increase robustness of additional investigation on this topic, larger databases should be considered. Finally, in line with the former point, our database was quite unbalanced towards subjects with protective genetic variants. This issue is partially due to the categorization of 2 of the 3 possible genotypes as protective (*e.g.* *CLU* TT and TC genotypes as protective and CC ones conferring risk). This criterion to classify individuals in each category resulted in unbalanced groups and may introduce some bias to our calculations that should be addressed enrolling new participants in future studies.

Our results showed that the brain activity from AD patients with risk alleles is characterized by a statistically significant decrease of RP and SE in beta band, in comparison with subjects with two protective alleles. Since these genetic elements are associated with alterations in biochemical pathways in neurophysiological patterns, the changes obtained in beta activity may have some relation with greater accumulations of A$\beta$ or neurotransmission abnormalities. However, further research is needed to improve our understanding of AD at the molecular level.
